# Combination therapy to checkmate Glioblastoma: clinical challenges and advances

**DOI:** 10.1186/s40169-018-0211-8

**Published:** 2018-10-16

**Authors:** Debarati Ghosh, Saikat Nandi, Sonali Bhattacharjee

**Affiliations:** Cold Spring Harbor Laboratory, Cold Spring Harbor, NY USA

**Keywords:** Combination therapy, *Glioblastoma multiforme*, Glioblastoma stem cells, Targeted cancer therapy, Tumor microenvironment, Precision medicine, Chemotherapy, Tumor heterogeneity, Drug resistance, Radiotherapy, Immunotherapy, Clinical trials

## Abstract

Combination therapy is increasingly becoming the cornerstone of current day antitumor therapy. Glioblastoma multiforme is an aggressive brain tumor with a dismal median survival post diagnosis and a high rate of disease recurrence. The poor prognosis can be attributed to unique treatment limitations, which include the infiltrative nature of tumor cells, failure of anti-glioma drugs to cross the blood–brain barrier, tumor heterogeneity and the highly metastatic and angiogenic nature of the tumor making cells resistant to chemotherapy. Combination therapy approach is being developed against glioblastoma with new innovative combination drug regimens being tested in preclinical and clinical trials. In this review, we discuss the pathophysiology of glioblastoma, diagnostic markers, therapeutic targeting strategies, current treatment limitations, novel combination therapies in the context of current treatment options and the ongoing clinical trials for glioblastoma therapy.

## Introduction

In recent years, a growing number of successful pre-clinical agents have failed to show reproducible effects on patient survival [1]. The “Basket trail” approach—testing the effect of one drug on a single mutation in a variety of tumor types is being used to address the irreproducibility issue [2]. Another approach being used to overcome this troubling trend is the use of combination of drugs that rely on complementary mechanisms of antitumor activity and can be combined into a therapeutic regimen [3, 4]. The success of combination therapy approach relies on three factors: (i) each component of the combination therapy regimen should have a single-agent activity without any cross-resistance, (ii) pre-clinical studies on the drug cocktail should indicate synergism, and (iii) each component should have a separate safety criterion [5]. Since, combination therapy works synergistically or in an additive manner, lower doses of individual drugs are administered reducing the problems of drug resistance to tumor cells and drug toxicity to healthy cells [6].

In cancer, combinations of two or more therapeutic treatments have been demonstrated to be more effective than monotherapy and chemotherapy [6]. Monotherapy non-selectively targets rapidly growing cells and chemotherapy leads to high toxicity burden and immunosuppression [6]. *Glioblastoma multiforme* (GBM) is the most frequent and aggressive brain tumor in adults with very poor prognosis [7]. In the past decade, despite novel therapeutic targets being discovered, monotherapy has failed in clinical trials [8, 9]. In this review, we discuss and summarize the current status of combination therapy in combating GBM.

## Pathophysiology of *Glioblastoma multiforme*

Glioblastoma multiforme is the most aggressive primary malignant brain tumor, with a median survival of about 14.6 months post-diagnosis and is classified as Grade IV by the World Health Organization (WHO) [10]. It accounts for 45.2% of malignant primary brain and central nervous system (CNS) tumors, 54% of all gliomas and 16% of primary brain and CNS tumors [11]. The global incidence of GBM is 0.59–3.69 per 100,000 live births with an age-adjusted incidence rate of 3.97 cases per 100,000 for males and 2.53 cases per 100,000 for females [11, 12]. Glioblastoma is classified into two types: primary GBM, which is the predominant subtype (80% of cases) and is manifested at later age (mean age 62 years) whereas secondary GBM progresses from lower grade astrocytoma or oligodendroglioma and is prevalent in younger patients (mean age 45 years) [13–16]. A rare subtype of GBM, “with oligodendroglioma component” (GBMO) has been added to the WHO classification [10]. GBMO is defined as GBM with an area resembling anaplastic oligodendroglioma and necrosis with or without microvascular proliferation [10].

### Molecular sub-classes of *Glioblastoma multiforme*

The histological homogeneity despite clinical heterogeneity of GBM sub-types that affect disease prognosis and therapeutic outcomes warrants a molecular profiling based classification [17]. The Cancer Genome Atlas (TCGA) summarized genomic alteration in 206 GBM patients. A total of 601 genes and sequencing data from 91 patients (a subset of the 206 GBM patients) were used to describe the mutational spectrum [18]. An independent set of gene expression profiling data from 260 GBM patients and 40 classifying genes have been used to cluster GBM into four clinically relevant subtypes; namely: Classical, Proneural, Mesenchymal and Neural (Table 1) [18].

**Table 1:** *Glioblastoma multiforme* classification by The Cancer Genome Atlas and World Health Organization

Sub-class	Genetic markers	Median survival (months)	References
Classical	CDKN2A, EGFR, NES, PTEN, Notch and SHH pathway	12.2	[18]
Mesenchymal	NF1, PTEN, TP53, TNF, NF-kB pathway	11.8	[18]
Proneural	PDGFRA, IDH1, PIK3A/PIK3R1, CDKN2A, PTEN, SOX, DCX, DLL3, ASL1, TCF4	11.3	[18]
Neural	EGFR, NEFL, GABRA1, SYT1, SLC12A5	13.1	[18]
Glioblastoma, IDH1 wild type	TERT, TP53, EGFR, PTEN	9.9	[21]
Glioblastoma, IDH1 mutant	TERT, TP53, ATRX	15	[21]

### Classical GBM

The classical subtype is associated with chromosome 7 amplification, loss of chromosome 10 (100% cases) and Epidermal growth factor receptor (EGFR) amplification (97% cases) [19]. Focal deletion of 9p21.3 harboring cyclin-dependent kinase Inhibitor 2A (CDKN2A) is associated (p < 0.01, two sided Student’s t test) with classical GBM, which co-occurs with EGFR amplification in 94% cases [18]. Homozygous deletion of 9p21.3 is almost mutually exclusive with retinoblastoma (RB) pathway genes (*RB1, CDK4, CCDN2*) [18]. Neural precursor and stem cell marker (NES) overexpression, mutation in phosphatase and tensin (PTEN), Notch (*NOTCH3, JAG1, LFNG*) and Sonic hedgehog (SMO, GAS1, GLI2) pathway activation are observed in this subtype [18].

### Mesenchymal GBM

This subtype is associated with focal heterozygous deletion of 17q11.2 containing neurofibromin 1 (NF1) (p < 0.01, adjusted two-sided Student’s t test), mutation in PTEN, mutation in tumor protein p53 (TP53) and activation of genes in tumor necrosis factor (TNF) superfamily and NF-kB pathway [18]. Mesenchymal subtype is associated with mesenchymal markers: *CHI3L1* and *MET* [18, 20].

### Proneural GBM

This subtype is associated with platelet-derived growth factor receptor alpha (PDGFRA) amplification, mutation in isocitrate dehydrogenase 1 (IDH1), PIK3A/PIK3R1, TP53, CDKN2A and PTEN [18]. Several development genes (SOX, DCX, DLL3, ASCL1, TCF4) are overexpressed in the proneural subtype [20]. Interestingly, 90% of the IDH1 mutations in GBM are found in proneural subclass (p < 0.01, adjusted two sided Fisher’s exact test) [18].

### Neural GBM

This subtype is associated with neuronal marker expression such as NEFL, GABRA1, SYT1, SLC12A5 and EGFR amplification and overexpression. The gene ontology (GO) categories associated with this subtype include neuron projection, axon transmission and synaptic transmission. This subgroup shows strong association with oligodendrocytic and astrocytic differentiation. The neural subgroup also includes genes that are differentially expressed during neuronal differentiation [18].

In 2016, WHO updated the classification of CNS tumors [21]. The new update includes molecular features in addition to histopathology [21]. The nomenclature of the subtypes includes the histopathological name followed by genetic information [21]. Currently, GBM is classification into three major sub groups (Table 1) [21].

### Glioblastoma, IDH1 wild type

About 90% of GBM are in this subtype, most cases are either primary or de novo glioblastoma [21]. Patients in this subtype are mostly 55 years or older [22]. The overall survival with surgery and radiotherapy is approximately 10 months. These tumors are located in supratentorial region of the brain and are associated with extensive necrosis, TERT promoter methylation (72% cases), TP53 mutations (27%), EGFR amplification (35%) and PTEN mutations (24%) [21]. A new variant, epithelioid glioblastoma, falls under IDH1-wild type and is characterized by the presence of large epithelioid cells, vesicular chromatin, rhabdoid cells and prominent nuclei [20, 21]. This subtype of GBM is found in patient ages ranging from 10 to 69 years and often harbors a v-raf murine sarcoma viral oncogenes homolog B1 (BRAF) V600E mutation [23]. This subtype lacks EGFR amplification and chromosome 10 loss [21].

### Glioblastoma, IDH1 mutant

About 10% GBM cases form this subtype and are characterized as secondary glioblastoma associated with low-grade diffused glioma [21]. This subtype of GBM arises in younger patients (about 44 years) [22]. The median survival post surgery and radiotherapy is 24 months. Additional chemotherapy treatment extends the medial survival to 31 months. These tumors are located in frontal lobe of brain and are characterized by limited tumor necrosis, TERT promoter mutation (26%), TP53 mutations (81%) and ATRX mutations (71%) [21].

### Glioblastoma, not otherwise specified (NOS)

This subtype includes GBM where full IDH evaluation cannot be performed [21].

There are two additional glioblastoma subtypes: (i) glioblastoma with primitive neural component and (ii) small cell glioblastoma and granular cell glioblastoma [21, 24, 25]. Both these subtypes show poor glioblastoma-like prognosis without microvascular proliferation and/or necrosis [21].

## Disease prognosis in *Glioblastoma multiforme* and clinical correlation

Many signaling pathways, oncogene and transcription factors are altered in GBM [26]. Disease prognosis in GBM and patient response to therapy depends on the type genetic and epigenetic modifications [27]. The following section summarizes the factors that determine GBM prognosis and impact its clinical management.

### Molecular marker based disease prognosis

Here we summarize the latest development in molecular marker based GBM prognosis and their clinical outcomes (Table 2).

**Table 2:** Molecular marker based disease prognosis in *Glioblastoma multiforme*

Markers	Genetic/epigenetic alteration	Pathway affected	Prognosis	References
MGMT	Promoter methylation	DNA mismatch repair	Better	[125]
EGFR	Gene mutation/partial deletion [EGFRΔIII]	PI3K/AKT/MAPK	Poor	[126]
IDH1	Point mutation [R132H]	G-CIMP and metabolic alteration	Better	[127]
G-CIMP	Hypermethylation	Global epigenetic alteration	Better	[18, 39, 40]
ATRX	Gene mutation	Alternative telomere lengthening	Poor	[45, 46]
TP53	Gene mutation	p53	Unknown	[42]
PTEN	Gene mutation	PI3K/AKT/MAPK	Poor	
1p19q deletion	CIC and FUBP mutation	Not clearly known	Better	[48]
SRC	Phosphorylation	Integrin signaling	Better	[49]
RPS6	Phosphorylation	mTOR	Poor	[49]

### *O*-6-methylguanine-DNAmethyltransferase (MGMT)

DNA repair enzyme MGMT removes alkyl groups from O6 position of guanine in DNA making cells resistant to the chemotherapeutic agent temozolomide (TMZ) [28]. Methylation of MGMT gene promoter leads to epigenetic silencing of MGMT in glioma cells. This in turn, leads to reduction in DNA repair function, increased genome instability and chemosensitivity to TMZ [29–32]. GBM patients with concurrent methylation of MGMT, TP53 and CDKN2A show better prognosis [33].

### EGFR

EGFR mutation is mainly found in primary GBM [34]. EGFR variant III (EGFRvIII) is the most common mutation (40%) in GBM and its overexpression is highly associated with poor prognosis [35]. EGFR gene status and overexpression is a prognostic indicator in younger patients [35].

### IDH mutation

Mutations in IDH1 or IDH2 genes, which encode isocitrate dehydrogenase enzyme involved in tricarboxylic acid cycle (TCA), are common in lower grade and anaplastic (II–III) glioma [36]. IDH1 mutated high grade gliomas arise from lower-grade glioma (secondary GBM) and shows specific radiographic, histologic and transcriptional features consistent with a less aggressive prognosis [37].

### G-CIMP

The CpG island hypermethylation phenotype (G-CIMP) is strongly correlated with IDH1 mutation and the proneural sub-type of GBM [38, 39]. G-CIMP is rarely found in primary GBM (5–8%) [39]. GBM patients in this subgroup have the highest overall survival rates [18, 39, 40].

### TP53 mutation

TP53 gene mutation is most commonly found in secondary GBM (60–70%) [41]. Mutation in this tumor suppressor is reported in younger patients, albeit the prognosis in this subtype is still unclear [42].

### ATRX mutation

Mutation in alpha-thalassemia/mental retardation syndrome X-linked (ATRX) gene, involved in alternative telomere lengthening is most frequently found in secondary GBM (57%), less frequently in pediatric GBM (24%) and rarely in primary GBM (4%) [43, 44]. This mutation often clusters with IDH1 and TP53 mutations and is associated with poor patient prognosis [45, 46].

### Loss of chromosome 10

Loss of chromosome 10, a part or the entire chromosome is found in 80–90% of GBM cases [41]. Mutation in PTEN, located at 10q23.3 is exclusively found in primary GBM and accounts for 20–40% of GBM [47].

### 1p19q co-deletion

GBMO results from 1p19q co-deletion [48]. As per the National Comprehensive Cancer Network (NCCN) treatment guidelines, 1p19q co-deletion is the only molecular biomarker for therapeutic use for GBMO subtype (NCCN, 2013).

### Metabolic profiling based prognosis

Protein expression analysis in GBM patients revealed two populations of glioblastoma stem-like cells (GSC) associated with different clinical outcomes: (i) proto-oncogene, tyrosine protein kinase and SRC activation is associated with the subgroup showing better prognosis (ii) ribosomal protein S6 (RPS6), an effector of the mTOR pathway is associated with poor patient prognosis [49].

### Immunological profiling based prognosis

Analysis of tumor-associated stroma revealed that mesenchymal (MES) subtype of GBM is associated with the presence of tumor-associated glial cells and microglial cells [50]. Furthermore, genetic deactivation of NF1 is associated with increased macrophage/microglia in the tumor microenvironment [50]. Since there is a higher frequency of M2 macrophages and CD4+ T cells observed after radiotherapy, M2 macrophages are presumed to play a role in radioresistance [50].

## *Glioblastoma multiforme:* treatment challenges

Despite the identification of well-defined molecular markers, therapeutic targeting and disease prognosis in GBM is poor. Multiple factors contribute to this disease complexity and are summarized below.

### Glioblastoma stem cell (GSC) and therapeutic resistance

The poor prognosis of GBM can be attributed to its resistance to current therapeutic approach consisting of radiotherapy (RT) with concomitant and adjuvant TMZ therapy post surgery [51]. Although, GBM is most comprehensively characterized based on genetic (IDH1 mutation), epigenetic (MGMT promoter methylation) and transcriptional (classical, mesenchyme, proneural, neural) profiling, these mutations show therapeutic resistance and recurrence due to the self-renewing tumor cell type known as GSCs that escape chemo-RT and proliferate residual tumor cells following treatment [52–54]. GSCs are defined as tumor cells capable of self-renewal, high tumorigenic ability, and capacity for multipotent differentiation [55, 56]. GSCs can acquire resistance to chemo-RT either through innate properties of genetic heterogeneity of the tumor or through adaptive resistance pathways [57–59].

### Intratumoral heterogeneity

Genomic landscape study of pre- and post treatment GBM patients pairs revealed a variable degree of genetically related clones to the original tumor (clonal evolution) and accumulation of new clones (sub clonal evolution)—giving rise to high degree of intratumoral heterogeneity: the primary cause for the poor prognosis and lack of effective therapeutic options in GBM [60, 61]. Intratumoral heterogeneity can be experimentally concluded from (i) single cell RNA-seq data showing the presence of heterogeneous mixture of cells in various GBM subgroups, and (ii) genetically distinct identity and differential drug resistance profiles in single cell derived GBM subclones [62, 63]. The proneural subgroup has the highest proportion of markers specific to other subgroups and reports the worst prognosis [62]. Altogether, intratumoral heterogeneity fuels therapy resistance and failure of effective therapeutic strategies.

### Post-therapy resistance

A number of molecular mechanisms have been implicated in the post-therapy resistance in GBM: DNA damage checkpoints; tumor microenvironment such as hypoxia, acidic and metabolic stresses; oncogene and transcription factor activation such as Notch, NF-kB and EZH2 [54, 64–71]. Some examples of these mechanisms include (i) enrichment of CD133+ GSCs post chemo-RT results in post-therapy resistance and is mediated by the activation of DNA damage checkpoint kinases (ChK1 and ChK2) [54], (ii) MGMT plays an important role in TMZ resistance mediated by MMR pathway that acts by reversing the mutagenic DNA lesion *O*-6-lguanine (introduced by TMZ) back to guanine [28, 72], (iii) the Notch pathway inhibitor: γ-secretase inhibitor (GSI) sensitizes GSCs to radiation [67] and overexpression of Notch 1 or Notch 2 sensitizes GSC to RT by blocking the transition into endothelial progenitors [67], (iv) combinations of receptor tyrosine kinase (RTK) inhibitors and/or RNA interference show promising anticancer activity by inhibiting cell growth in PTEN-deficient glioma cells [73], (v) hypoxia favors GSCs maintenance and hypoxia inducible transcription factors (HIF-1 and HIF-2) are involved in tumor maintenance and angiogenesis [74], and (vi) GSCs suppress the adaptive immune system by recruiting microglia/macrophages to induce secretion of immunosuppressive cytokines interleukin-10 (IL-10) and TGF-β1, in turn promoting tumor growth [75].

## Therapeutic targeting

The compromised responses to radio and chemotherapy in GBM results from therapeutic resistance and inefficient targeting of GSCs. Novel therapeutic approaches are being developed to overcome these treatment limitations [76–80].

### Targeting GSCs by surface markers

Patients with CD133+ expression show poor clinical outcomes [81]. Silencing of CD133+ in GBM derived neurosphere impairs the self-renewal and tumorigenic capacity of neurosphere cells [82]. Selective targeting of CD133+ GBM cells by anti-CD133 monoclonal antibody selectively kills tumor cells while sparing normal cells [83]. Using CD133 antibody conjugated immune liposomes that encapsulate gemcitabine to target GSCs showed 15 times higher anti-tumor effect than that of free gemcitabine [84].

### Targeting GSCs by signaling pathways

Signaling pathways including Notch, Sonic-hedgehog (Shh), VEGF, STAT3 and Bone morphogenetic proteins (BMPs) are critical in GSCs maintenance and targeting these pathways have promising therapeutic potential [85]. GSI mediated Notch inhibition leads to reduced neurosphere proliferation, reduced CD133+ cell fraction in vitro and decreased tumor growth in vivo [86]. STAT3 is a critical signaling node involved in GSC maintenance through regulation of Toll-like receptor TLR9 expression [87, 88]. A variant of BMP7 (BMP7v), member of the TGF-β superfamily, has been shown to decrease GSC proliferation and angiogenesis providing a novel approach to the treatment of GBM [89].

### Targeting tumor microenvironment

Tumor microenvironment plays a significant role in GSC stemness and thus targeting microenvironment for therapy has shown promising results [90]. Cancer stem cells (CSCs) orchestrate vascular niches that maintain the CSCs pool [74]. VEGF is involved in microvasculature formation and is an important mediator of angiogenesis in GBM [90]. Bevacizumab is a recombinant humanized monoclonal antibody that inhibits VEGF signaling pathway by inhibiting interaction with VEGF receptors [91]. Tumor-associated macrophages (TAMs) are enriched in GBMs and promote tumor progression [92]. GSCs secrete periostin (POSTN) to recruit TAMs and disrupted POSTN attenuate the tumor-supportive M2 type TAMs in xenografts [92].

## Combination therapy in *Glioblastoma multiforme*: preclinical development

Characterization of molecular targets from in vitro and in vivo studies have led to the development of clinical trials in GBM. Experimental models and clinical trials are most rewarding when they combine multiple gene targets [93–96]. This approach, also referred to as combination therapy, shows synergistic effects in terms of drug efficacy and is by far the most effective way of managing aggressive tumors like GBM.

**Fig. 1:**
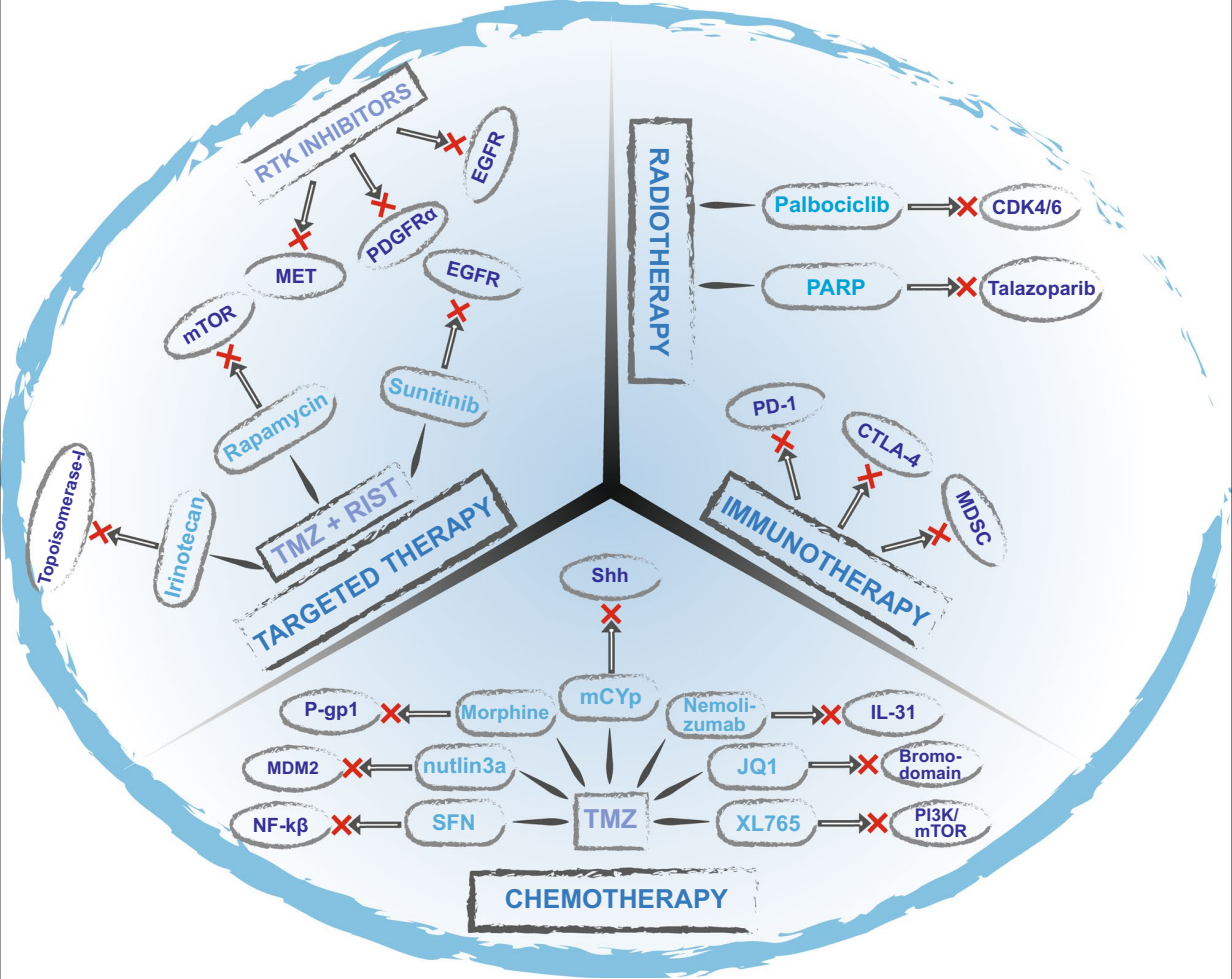
Schematic representation of combination therapy in treatment of GBM. Targeted therapy approaches have the potential to improve patient outcome in the treatment of Glioblastoma. Small-molecule inhibitors in combination with chemo/radiotherapy can overcome chemo/radioresistance in patients improving treatment efficacy when compared to monotherapy. We summarize small molecule inhibitors and their gene targets that are in preclinical development for GBM therapy and are being tested in combination with radio- and/or chemotherapy

Maximum surgical resection of the tumor followed by focal chemotherapy with TMZ is the current standard of care for GBM [97]. TMZ, a second-generation imidazotetrazine, is a DNA-alkylating agent [98]. It has the ability to cross the blood–brain barrier (BBB) making it particularly effective in the treatment of brain tumors [98–100]. However, in addition to severe side effects of TMZ such as myelotoxicity, ulcers, nausea, vomiting, fatigue and toxic DNA damage, the resistance to this drug is common in GBM patients [101, 102]. A potential approach in the first-line treatment of GBM may be to explore a more effective combination regimen. In this section we summarize a comprehensive list of gene targets and small molecule inhibitors that are in various stages of preclinical development for GBM therapy and are being tested in combination with radio- and/or chemotherapy (Fig. 1).

### TMZ in combination therapy

#### TMZ and micellarized Cyp (MCyp)

Shh signaling pathway is up regulated in high-grade gliomas and inhibition of Shh leads to apoptosis and cell death [103, 104]. Cyclopamine (Cyp) is a Shh antagonist, which is selective and non-toxic to cell types not dependent on activation of the Shh pathway [105]. Combination of MCyp with TMZ has been shown to block the Shh pathway and eliminate neurosphere formation [105].

#### TMZ and morphine

The efflux of P-glycoprotein 1 (P-gp1) in endothelium cells of the BBB mediates TMZ resistance [106]. Morphine is an inhibitor of P-gp1 [106]. Combinatorial effect of morphine with lower dose of TMZ shows significant reduction in tumor growth [106]. Additionally, reducing the TMZ drug dose reduces chemoresistance, thus improving therapeutic response in the long term [106].

#### TMZ and nutlin3a

nutlin3a is a murine double minute 2 (MDM2) protein–protein interaction inhibitor [107]. It blocks the MDM2-p53 associated signaling pathways [107]. The combination of TMZ/nutlin3a synergistically decreases the growth of p53 wild type GBM cells and leads to significant increase in survival of mice with GBM10 intracranial tumor [107].

#### TMZ and SGT-53

Combining TMZ with exogenous delivery of TP53 via tumor-targeted nanocomplex (SGT-53) significantly chemosensitized GBM cells (U87 and U251) to chemotherapy and prolonged median survival [108].

#### TMZ and sulforaphane (SFN)

Coadministration of the transcriptional NF-kB inhibitor, SFN with TMZ in TMZ-resistant cell lines (U87-R and U373-R) lead to reversal of chemoresistance, suppression of cell growth and enhanced cell death in chemoresistant xenograft in nude mice [109]. Additionally, SFN has also been shown to inhibit miR-21 via Wnt/β-catenin/TCF4 signaling pathway, thereby enhancing chemosensitivity [110].

#### TMZ and XL765

The PI3K/mTOR pathway is dysregulated in many tumors. It acts by inhibiting Akt signaling and promotes resistance to EGFR inhibitors [111, 112]. XL765 is a novel PI3K/mTOR dual inhibitor [113]. XL765 in combination with TMZ shows additive cytotoxicity in genetically diverse GBM xenografts [113].

#### TMZ and JQ1

Delivery of transferring-functionized nanoparticle (Tf-NP) based dual drug combination of TMZ with bromodomain inhibitor JQ1 leads to increased DNA damage and apoptosis. This results in 1.5 to 2-fold decrease of tumor burden and increased survival when compared with free-drug dosing [114].

#### TMZ and nimotuzumab

The mutant form of EGFR, EGFRvIII confers therapeutic resistance and tumor growth. Nimotuzumab, an anti-EFGR antibody in combination with TMZ showed enhanced antitumor activity in nude mice bearing subcutaneous or intracerebral tumor expressing EGFRvIII [115].

### RIST/aRIST in combination therapy

The RIST (rapamycin, irinotecan, sunitinib, temozolomide) and the variant aRIST (alternative to rapamycin, GDC-0941) combination regimen inhibit GBM cell growth in primary patient culture via up-regulation of apoptotic pathways [116]. Rapamycin is an mTOR inhibitor, irinotecan is a topoisomerase-I inhibitor and sunitinib inhibits RTKs [116]. These individual components have shown partial success, but while in combinations, they significantly reduce cell viability [116]. Additionally, in the combinatorial regimen all inhibitors are administered at relatively lower doses thereby reducing toxicity and other side effects [116].

### Radiotherapy in combination therapy

#### RT and poly (ADP ribose) polymerase (PARP) inhibitor

Inhibition of PARP proteins radiosensitizes glioma cells by inhibiting DNA repair [93–95]. Studies show that PARP inhibitors (PARPi) decreased colony formation in MGMT unmethylated GBM patient derived xenografts. This suggests PARP inhibition as a new therapeutic approach in GBM [117]. RT in GBM patients’ lead to upregulation of PARP1 mediated repair of DNA damage in glioblastoma CSCs. Preclinical study of the PARPi, talazoparib (BMN-673; Pfizer), in combination with RT showed prolonged G2/M block and a significant reduction in GSC proliferation [118].

#### RT and palbociclib

RB pathway (Cycline-dependent kinase (CDK4/6), RB tumor suppressor and the E2F-family of transcription factors are important for cell cycle regulation [119]. Amplification of CDK6 and deletion of CDK inhibitor 2A/B (CDKN2A/B) genes are frequently found in GBM patients (~ 86%), specifically in classical and mesenchymal subtypes [119]. Such alterations cause constitutive expression of E2F transcription factors leading to cell cycle acceleration, DNA replication and mitotic progress [119]. Palbociclib (PD0332991; Pfizer) is a selective inhibitor of CDK4/6 kinase in RB proficient cells and can promote cell cycle arrest and apoptosis both in vitro and in vivo [120]. Palbociclib when coadministered with RT, showed a survival advantage in mice [121].

### Tyrosine kinase receptor inhibitors in combination therapy

Activation of RTKs, including EGFR, PDGFRα, PDGFRβ and MET is frequently observed in GBM. Anti-EGFR therapy or targeted therapy against a particular RTK is not effective in suppressing the downstream PI3K pathway leading to therapeutic resistance. Combination of RTK inhibitors (erlotinib, SU11274, imatinib) show improved cell survival and anchorage-independent growth in PTEN-deficient glioma cells [73].

### Immunotherapy in combination therapy

Immunosuppression is one of the primary reason for poor prognosis in GBM [122]. Reduction in T cell mediated immune response is due to co-inhibitory receptors on T-cells known as immune checkpoint molecules [122]. CTLA-4 and PD-1 are two such immune checkpoint molecules, blocking these two molecules induces tumor regression and promotes long-term survival [123, 124]. Combination of anti-PD-1 antibodies and RT doubled median survival and enhanced long-term survival in 15–40% GBM mice [125]. Studies in GBM models and human samples have shown that the accumulation of myeloid-derived suppressor cells (MDSCs) in the tumor microenvironment induces immunosuppression [126, 127]. Inhibiting MDSC accumulation with PD-1 or CTLA-4 (that acts early in T-cell activation) enhances efficacy of immune-simulated gene therapy [128].

### Antisense oligo-based therapy in combination therapy

Immune-suppression in the tumor microenvironment leads to down-regulation of TGFβ-2 in GBM cells, which inhibits T and B cell activation and proliferation. Nanoparticle mediated delivery of antisense oligonucleotides (AON) to GBM xenografts in rats showed better delivery of AON to target cells and increased rates of activation of CD25 + T cells leading to immunostimulation [129, 130].

## Combination therapy in GBM: ongoing clinical trials

Molecular profiling of GBM and preclinical research has led to the discovery of several targets for GBM therapy. The success of poly (ADP ribose) polymerase inhibitors (PARPi) in the treatment of breast and ovarian cancer in recent years has led to an intensive focus on exploiting the underlying combination therapy approach for both discerning the mechanisms of oncogenesis as well as for developing better treatment regimens. Depending upon the GBM sub-type, treatment modalities that combine two or more chemotherapeutic agents are being tested in ongoing clinical trials. In this section we summarize a list of the ongoing clinical trials for various GBM targets that are being tested in combination with radio-and/or chemotherapy (Table 3).

**Table 3:** Combination therapy in clinical trials for treatment of *Glioblastoma multiforme*

Target	Molecule	GBM type	Stage of testing	References
Alkylation mediated DNA damage	Temozolomide (TMZ) + radiotherapy (RT)	GBM	Phase IV	NCT00686725
αvβ3 and αvβ5 integrin inhibitor+	Cilengitide + TMZ + RT	Newly diagnosed GBM patients with methylated MGMT promoter	Phase III	NCT00689221
Tyrosine kinase inhibitor	Imatinib mesylate + hydroxyurea	TMZ resistant progressive GBM	Phase III	NCT00154375
Pan-VEGFR tyrosine kinase inhibitor	Cediranib + lomustine chemotherapy	Recurrent GBM	Phase III	NCT00777153
VEGF-A	Bevacizumab (Avastin^®^) + TMZ + RT	Newly diagnosed GBM	Phase III	NCT00943826
Immunostimulant	TMZ + RT + poly ICLC	Newly diagnosed GBM	Phase II	NCT00262730
Multiple kinase inhibitor	TMZ + RT + sorafenib	GBM	Phase II	NCT00544817
Tubulin inhibitor	TMZ + PPX (CT2103)	GBM without MGMT methylation	Phase II	NCT01402063
mTOR inhibitor	TMZ + RT + bevacizumab + everolimus	GBM	Phase II	NCT00805961
VEGF-A + topoisomerase I inhibitor	TMZ + avastin + irinotecan	Unresectable/Multifocal GBM	Phase II	NCT00979017
EGFR inhibitor	TMZ + bevacizumab + tarceva	GBM	Phase II	NCT005255525

## Future perspectives

Recent therapeutic approaches in cancer are based on the major advances made in areas of molecular biology, cellular biology and genomics. Administering drug combinations to patients that provide better clinical outcomes than individual agents do, is one such advance that has been successfully translated into therapy. PARP inhibitors, which utilize the combination therapy approach, have shown excellent results in the treatment of breast and ovarian cancer changing the diagnostic and therapeutic landscape of the disease. Since combination drugs target multiple pathways, they rely on smaller drug doses, which help minimize drug resistance, a pitfall of adjuvant therapy in the clinic. However, it is important to consider some limitations to the use combination therapy in GBM. First, a better understanding of the signaling networks and molecular players in GBM is crucial for the development of combination regimens and the success of this approach. Second, some drug combinations are effective and act synergistically for therapeutic benefits; some drug interactions may produce unwanted side effects on the patient’s health. Tumor heterogeneity and the unique immunological milieu of CNS is a major challenge in designing effective therapy regimens for GBM. One approach to overcome these limitations is to build mathematical models of synergism/antagonism of drugs and pathways that are effective in predicting drug combinations for multifactorial disorders like GBM. Mathematical models are effective in visualizing drug combinations in a dose response matrix that can be subsequently validated by in vitro and/or in vivo experiments [131]. Quantification of synergism (when the result of combining two or more chemical compounds produces an effect that is greater than additive effects of individual compounds) or antagonism (when the result of combining two or more chemical compounds produces an effect that is less than the additive effects of the individual compounds) is assessed based on the deviation of the observed combination response from the expected combination response calculated using a reference model [132]. The most commonly used reference models for calculating combination drug response include the Highest single agent (HSA) model, Loewe additivity model, Bliss independence model, and the Zero interaction potency (ZIP) model [133–136]. Combination therapy and drug synergism for targeted heterogeneous tumors and the interacting tumor microenvironment thus holds promise. Future research should focus on identifying synergistic interactions between chemotherapy, radiotherapy and immunotherapy in order to maximize the antitumor potential of individual treatment approaches.

## Abbreviations

ATRX: alpha-thalassemia/mental retardation syndrome X-linked
aRIST: alternative to rapamycin, GDC-0941
AONs: antisense oligonucleotides
BMP: bone morphogenetic protein
CSCs: cancer stem cells
CNS: central nervous system
G-CIMP: CpG island hypermethylation phenotype
CDK: cycline-dependent kinase
Cyp: cyclopamine
EGFR: epidermal growth factor receptor
GSI: γ-secretase inhibitor
GBM: glioblastoma multiforme
GBM-O: glioblastoma multiforme with oligodendroglial component
GSC: glioblastoma stem-like cells
HIF: hypoxia inducible transcription factors
IL: interleukin
IDH1: isocitrate dehydrogenase 1
mCyp: micellarized Cyp
MMR: mismatch repair
MDM2: murine double minute 2
MDSCs: myeloid-derived suppressor cells
NF1: neurofibromin 1
MGMT: *O*(6)-methylguanine-DNAmethyltransferase
PARPi: PARP inhibitors
POSTN: periostin
PTEN: phosphatase and tensin
PI3K: phosphatidylinositol 3-kinase
PDGFRA: platelet-derived growth factor receptor alpha
RT: radiotherapy
RIST: rapamycin, irinotecan, sunitinib, temozolomide
Shh: Sonic-hedgehog
TMZ: temozolomide
TCGA: The Cancer Genome Atlas
Tf-NP: transferring-functionized nanoparticle
TAMs: tumor-associated macrophages
TNF: tumor necrosis factor
TP53: tumor protein p53
VEGF: vascular endothelial growth factor
